# Therapeutic monitoring of TNF inhibitors for rheumatoid arthritis: evidence required following NICE’s recommendations

**DOI:** 10.1093/rap/rkaa023

**Published:** 2020-06-22

**Authors:** Sean P Gavan, Meghna Jani, James Bluett, Katherine Payne, Anne Barton

**Affiliations:** r1 Manchester Centre for Health Economics, Division of Population Health, Health Services Research and Primary Care, School of Health Sciences, Faculty of Biology, Medicine and Health, The University of Manchester; r2 Division of Musculoskeletal and Dermatological Sciences, Centre for Epidemiology Versus Arthritis, Centre for Musculoskeletal Research, School of Biological Sciences, Faculty of Biology, Medicine and Health, The University of Manchester; r3 NIHR Manchester Biomedical Research Centre, Manchester University NHS Foundation Trust, Manchester Academic Health Science Centre; r4 Centre for Division of Musculoskeletal and Dermatological Sciences, School of Biological Sciences, Faculty of Biology, Medicine and Health, The University of Manchester, Manchester, UK

Immunogenicity to TNF inhibitors (TNFi) can affect drug pharmacokinetics and response to treatment [1]. Drug level and anti-drug antibody (ADAb) testing, also known as therapeutic drug monitoring (TDM), for people receiving TNFi treatment has been advocated across different chronic inflammatory diseases [1]. The information from each test, in isolation or in combination, can aid prescribing decisions by informing the feasibility of dose tapering or by indicating the next-line treatment after (primary and secondary) non-response. Current evidence supporting the clinical effectiveness and cost-effectiveness of TDM has become the focus of leading professional bodies for rheumatology [2]. In July 2019, the National Institute for Health and Care Excellence (NICE) concluded that there was insufficient evidence to recommend TNFi drug level and ADAb testing by ELISA routinely for RA and that further research was needed [3]. The recommendation by NICE, as part of the Diagnostics Assessment Programme, was the product of a 16-month assessment and appraisal process underpinned by deliberation of supporting clinical and economic evidence. Economic evidence is essential to decision-making by NICE and informs whether testing is a potentially cost-effective use of finite health care resources [4]. This editorial aims to provide an overview of how the recommendation by NICE was determined and to highlight implications for subsequent studies of TNFi ADAb and/or drug level testing in RA.

**Fig. 1:**
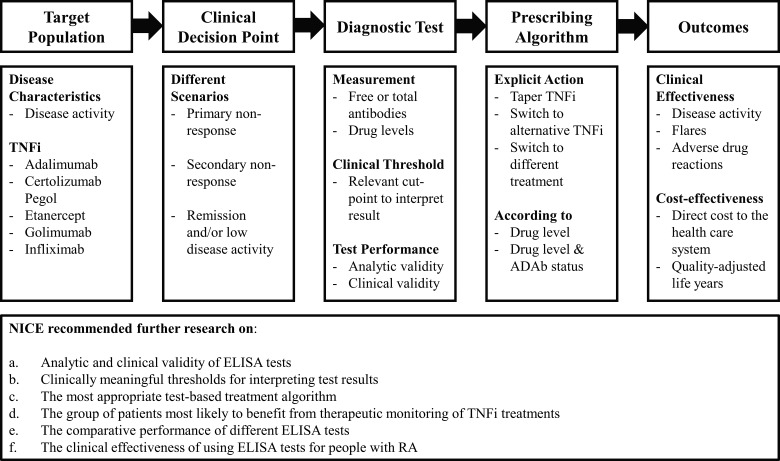
End-to-end evidence and considerations for further research by the NICE Diagnostics Assessment Programme These recommendations were summarized from *NICE Diagnostic Guidance 36* [*3*]. ADAb: anti-drug antibody; TNFi: TNF inhibitor.

The economic evidence used by NICE comprised a decision-analytic model-based cost-effectiveness analysis that compared alternative courses of action in terms of their costs (to the health care system) and health consequences [measured using quality-adjusted life years (QALYs)] [5]. Alternative courses of action in this context were management strategies that included TNFi drug level and ADAb testing and current practice without testing. Understanding the economic impact of a testing strategy requires ‘end-to-end’ evidence (Fig. 1) that demonstrates how information from a test informs clinical management and, in turn, how this change in management affects cost and health outcomes [6]. NICE defines relative cost-effectiveness within its Diagnostics Assessment Programme as a ratio of incremental costs to incremental QALYs below a range of £20 000 to £30 000 per QALY gained [4].

The scope of the assessment by NICE included three clinical decision points for people with RA who received different TNFi treatments and /firhad reached their treatment target (remission or low disease activity), not responded to TNFi treatment (primary non-response) or stopped responding to TNFi treatment (secondary non-response).

Five commercial ELISA kits (Promonitor; IDKmonitor; LISA-TRACKER; RIDASCREEN; MabTrack) and one test service (Sanquin Diagnostic Services ELISA) were identified to be relevant to the assessment [3]. These tests were appraised individually owing to differences in their characteristics (for example, unit cost and whether free or total antibodies were measured). A systematic review of clinical evidence identified studies that reported the decision impact (proportion of patients with treatment modification) and clinical utility (effect on clinical outcomes) of each ELISA at each clinical decision point [3].

The availability of clinical evidence presented the first significant barrier to the assessment. Only two studies (reported in different conference abstracts and full-text publications), the non-randomized INGEBIO trial [7] and a single-centre observational study [8], were identified, and both were of patients who had reached their treatment target. As a result, the diagnostics advisory committee were not provided with evidence to assess the cost-effectiveness of TNFi TDM for primary or secondary non-response [3]. Evidence was identified only for the Promonitor ELISA in patients who had reached their treatment target, which meant that data were not available to produce economic evidence for the other ELISA tests. The base-case result of TNFi TDM for people who had reached their treatment target, compared with current practice, exceeded the conventional threshold for cost-effectiveness used by NICE (£51 929 per QALY gained for people in remission and £125 272 per QALY gained for people in low disease activity or remission) [3]. Subsequent analyses suggested that these results were highly sensitive to the frequency and cost of testing [9, 10].

Sensitivity analyses undertaken for the NICE assessment indicated scenarios where testing might be cost-effective. However, the quality and relevance of the clinical evidence were questioned by the diagnostics advisory committee. For example, with respect to quality, the INGEBIO trial [7] was judged to be at serious risk of bias owing to an imbalance in disease activity between arms at baseline. The single-centre observational study [8] provided some support that ELISA testing could inform tapering without adverse outcomes but was limited by the absence of a control group. With respect to relevance, both studies included patients with diseases other than RA. Rates of immunogenicity might differ between rheumatic diseases, which contributed uncertainty about whether the findings of these studies applied to a population with RA only [3]. The clinical studies were undertaken in Spain, and the committee questioned whether the results could be generalized to a UK setting. For example, TNFi tapering is undertaken in Spain but not routinely in the UK. A prescribing algorithm to inform how test results should guide management decisions was also not reported by the INGEBIO study [3]. These concerns ultimately reduced the reliability of the base-case result from the economic analysis and its usefulness to inform decision-making.

The assessment and appraisal by NICE demonstrated how the availability, quality and relevance of clinical and economic evidence were barriers to recommending routine TNFi ADAb and drug level testing for RA. The guidance recommended that audit data should be collected if TDM is being undertaken currently for TNFi-treated RA patients [3]. Considerations for primary and secondary research (Fig. 1) were suggested by the guidance to help generate end-to-end evidence that will demonstrate how information from TNFi drug level and ADAb testing at different clinical decision points informs management decisions and longer-term outcomes. Crucially, this future research should include a prospective randomized study that compares TDM with current practice for people with RA in the UK, using clinically relevant ELISA tests and an explicit prescribing algorithm to inform management decisions, alongside decision-analytic modelling to extrapolate long-term health outcomes and costs to the health care system.

*Funding*: No specific funding was received from any bodies in the public, commercial or not-for-profit sectors to carry out the work described in this article.

*Disclosure statement:* M.J. was a member of the External Assessment Group for the NICE Diagnostics Assessment DG36: ‘Therapeutic Monitoring of TNF-inhibitors in Rheumatoid Arthritis’, and has received speaker’s fees for a scientific meeting by Grifols and travel expenses from AbbVie. J.B. has received a research grant award from Pfizer and travel bursaries from UCB and Pfizer. K.P. and M.J. are members of the EULAR Taskforce on Therapeutic Drug Monitoring of Biopharmaceuticals in Rheumatology. A.B. was a specialist committee member for the NICE Diagnostics Assessment DG36: ‘Therapeutic Monitoring of TNF-inhibitors in Rheumatoid Arthritis’. S.P.G. has declared no conflicts of interest.
